# The survival of survival auditions: The effects of cultural memes in the Korean TV broadcasting industry

**DOI:** 10.1371/journal.pone.0318193

**Published:** 2025-03-05

**Authors:** Doyoon Kim, Dongyoub Shin

**Affiliations:** 1 Division of Business Administration, Yonsei University, Wonju, Korea; 2 School of Business, Yonsei University, Seoul, Republic of Korea; Rikkyo University: Rikkyo Daigaku, JAPAN

## Abstract

This study empirically analyzes the evolution of cultural products based on theoretical cultural discourse and evolutionary processes. We use data from 116 survival auditions aired in Korea between 2006 and 2017 to examine the cultural memes that shape the continued appeal of survival audition programs. Specifically, we discuss the influence of “memes” in cultural codes, namely, audience empowerment, experts’ involvement, fair rewards, and career opportunities. The results of probit regression analysis with survival audition program reproduction as the dependent variable show that audience empowerment, experts’ involvement, fair rewards, and career opportunities in survival audition programs influence the reproduction of cultural goods. The findings confirm all four hypotheses. The findings of this study have theoretical and practical implications. First, it enriches the theoretical discourse on the evolution of cultural goods by offering a meme-based explanation for their reproduction. Second, it has implications for industry practitioners involved in planning and producing cultural goods by identifying normative cultural codes that affect the longevity of these products.

## Introduction

This study investigates the evolutionary dynamics of cultural goods by focusing on the influence of memes [1]. We attempt to explain why some cultural goods become very popular or run for multiple seasons, while others quickly disappear. We do this by empirically analyzing how cultural memes affect the survival rates of survival audition programs in the Korean TV broadcasting industry. Originating as star search programs, many variations of survival audition shows are now produced globally. Some programs have successfully run for over a decade, while others failed to survive even one season or vanished after a few episodes. This study explores the evolutionary forces behind the variations in survival rates from the perspective of cultural memes.

Studies have defined cultural goods [2–5] as the creation, industrial reproduction, and mass distribution of cultural works [6–8]. Most studies in this area have explored macro-level aspects of the cultural industry, such as industrial structure, competition, market conditions, and regulations that influence the output, such as music, broadcasting, film, publishing, design, and advertising [2,3,4,9–13]. In contrast, micro-level studies have focused on the production process and examined elements, such as production teams, performers, and genres [12,14].

Research on the success of cultural goods has often synthesized macro- and micro-perspectives to understand how cultural products gain market traction. While many studies have examined the success of movies and music based on industrial and internal factors, similar research on the “survival audition” format is relatively limited. However, recent studies have examined the popularity of survival audition programs [15–18].

Studies on sequels of cultural goods primarily focus on market-specific factors, such as the success of the original product or its potential based on similarities to the original series [18–21]. Unlike the film industry, where box office numbers provide a clear indicator for the production of a sequel, assessing the success of TV productions is more challenging because broadcasters and production companies withhold information regarding merchandise sales, advertising revenue, and licensing profits. Thus, this study examined the continued appeal of cultural goods by considering market-specific, cultural, and normative factors, such as “memes” that influence the success and longevity of cultural goods.

Our analysis centers on “cultural memes” embedded in cultural goods. Each good contains multiple memes in various combinations. Richard Dawkins [1] introduced the concept of memes in The Selfish Gene, likening memes to cultural counterparts of biological genes. Although internet memes, i.e., images, videos, or texts shared online, are derived from this concept, they have evolved into something that has a narrow focus—viral dissemination. This study returns to the original theoretical foundation of memes and defines and discusses them in their conventional sense.

This study examines the evolutionary dynamics of cultural goods, treating each as a carrier of multiple memes. Like genes diffusing through biological channels, memes spread among cultural goods through social mechanisms, which is primarily replication by imitation. Memes contained in cultural goods that persist are more likely to survive than other memes. According to the isomorphism argument of neo-institutionalism theory [22] and density dependence theory of organizational ecology [23], adopting practices with legitimacy enhances organizational survival. Similarly, we hypothesize that memes with specific characteristics increase the chances of survival of cultural goods. The focus of this study is identifying the memes that increase or decrease the prospects of survival of cultural goods.

Survival audition shows have a particular place in our attention, so we spent the time with those. Firstly, these programs are about competition and participants, so within them are themes that universally appear and vibrate with all audiences. The audience is interested in whether the contestants are treated in a fair way or not. On the other hand, survival audition shows have gained worldwide recognition. Native to the USA, these productions are now being seen in many places such as the Netherlands, Germany, South Korea, and China. Fourthly, these events have branched into other areas of entertainment like music, fashion, cuisine, entrepreneurship, and acting which are the diversification that makes them possible to foreign cultures as well. At the end, survival auditions without reference to a particular platform are available wherever media happens. They were initiated during radio programs in the 1940s and later on made a switch to television. Their flexibility enabled them to stay connected and engage with different media platforms and consumer segment.We first identified and defined cultural memes in survival audition programs using content analysis. Next, we statistically estimated how these memes influence a program’s chances of survival into the next season. Specifically, we examined the effects of four cultural memes, namely, audience empowerment, experts’ involvement, fair rewards, and career opportunities, on the continued appeal of 116 Korean survival audition programs that aired between 2006 and 2017. We found that all four memes significantly affect the survival prospects of the shows containing them.

This study makes the following contributions. First, it highlights the need to implement tailored strategies based on the type of cultural goods, emphasizing creativity as the competitive advantage in the early-stage development of a show. Indeed, broad audience acceptance is essential for cultural goods to be repeatedly produced, such as survival audition programs. Second, the study introduces a meme-based approach to analyze cultural goods, offering a new framework that explains industry-wide evolutionary patterns, which conventional approaches focusing on individual product characteristics fail to capture. Last, it explores the relationship between abstract norms, particularly fairness, and their tangible expressions in evaluating cultural products. It provides new insights into how perceptions of fairness influence the success and dissemination of cultural products.

## Theoretical background

The 21st-century business environment is often called the “creative economy,” in which innovation has replaced efficiency in volume as the primary driver of economic growth [24,25]. As a result, cultural industries have attracted increasing attention from various disciplines [4,26–31]. However, the systematic study of the evolutionary dynamics of cultural goods remains underexplored; thus, it is necessary to clarify the key components of the term “cultural industry,” particularly goods.

This study explored the evolutionary dynamics governing the survival and continued appeal of cultural goods through the lens of cultural memes. We argue that specific cultural codes, or memes, associated with cultural goods have a considerable effect on their survival and continued appeal. We empirically identified cultural memes in survival audition TV programs using content analysis and evaluated how these memes affect the chances of survival of such programs.

Evolutionary theory scholars have proposed various units of selection, such as individual, group, kin, and gene [32]. According to gene selection theory, in biological evolution, the primary unit of selection is not the individual organism but the genes it carries [33]. Similarly, according to meme selection theory in cultural evolution, memes are “units of cultural transmission or imitation” [1, pp.192].

### Related concepts of meme and meme-based explanations

Memes represent the modes of thoughts, values, beliefs, and assumptions that are embedded within cultural goods [1,34]. As the cultural equivalent of genes, memes are units of information that are stored in a person’s brain, replicating themselves as the person observes and interprets cultural expressions [35]. Since Dawkins introduced the meme concept in 1976, many scholars have expressed concerns about its definition, underlying assumptions, and empirical measurability. The “social code” concept in organizational ecology similarly describes behavior patterns expected by audiences.

However, explaining cultural evolution using memes, instead of assumptions, values, or beliefs, has distinct advantages. First, there are nuanced differences between terms, such as assumptions, values, and beliefs [36–38]. Limiting culture to values or sense-making neglects its practical elements. Memes serve as an umbrella term for all cultural modes of thought [35] and benefit interdisciplinary research in philosophy [39], psychology [34], anthropology [40], and law [41]. Second, memes are valuable for their specificity; they distinguish modes of thought from their external forms, such as speech or actions [42].

This study explores the evolution of cultural goods through a meme-based explanation, focusing on the reproduction of specific goods over multiple rounds of selection. Survival audition programs have garnered high viewer engagement [43,44]. Unlike films or theater, these programs rely on cultural themes, such as transparency, authenticity, and interaction, and viewers directly participate through voting [44]. The notion of citizenship in viewer involvement has drawn considerable political and sociological attention, extending its influence beyond the cultural industry [45,46].

This study examines the continued appeal of survival audition programs in an evolving broadcasting landscape by focusing on how shifts in time, format, and channels influence this genre of programming. It explores the role of specific cultural codes embedded in cultural goods and their effect on the success of such type of shows. This study contributes to discussions in management, cultural sociology, broadcasting, and journalism by empirically analyzing the internal characteristics of shows and audience-related factors that shape the continued appeal of survival audition programs.

### Fairness meme in the survival of survival audition programs

The importance of memes varies depending on the characteristics of each cultural entity; hence, identifying the key meme is critical for the survival of cultural goods. Although one meme may decisively influence the survival of a particular cultural entity, it may not have the same effect on others. Therefore, a meme-centered approach prioritizes the identification of the distinctive combination of memes within individual cultural entities rather than focusing on universal memes present in all cultural goods. This study identifies the fundamental memes intrinsic to survival audition programs. We build on Levitt and March’s [47] argument on organizational routines by proposing that historical contexts deeply shape memes. Understanding the core memes within survival audition programs requires taking a comprehensive look at their historical evolution [47].

Survival audition programs first started in the 1940s, when radio broadcasters in the U.S. began producing talent competitions. The early format was simple; talent scouts recruited individuals who competed publicly in a studio, with a live audience voting for the winner. Until the mid-1970s, these talent competitions lasted only a season or two [48]. *The Gong Show* (1976–1980) changed the format by innovatively featuring celebrity judges, which enhanced the entertainment value and credibility of the show. The next key innovation came with multi-round competitions in *Star Search* (1983–1995), in which contestants competed across diverse genres, facing elimination based on audience’s and judges’ evaluations. *Star Search* became the prototype for later global formats, such as *Got Talen*t and *American Idol*.

Because of radio’s limitations, initially, music-based survival audition programs dominated this genre of programming. However, with the spread of television, survival auditions expanded into diverse sub-genres, incorporating audio and visual elements. One notable example is *Project Runway*, which used the survival audition format in fashion design from 2004 to 2017. Every week, contestants competed under various constraints, highlighting the growing genre diversity of survival audition programs.

Our analysis of Korean TV survival audition programs reveals the centrality of the fairness norm, a universally emphasized societal value, to the success of these programs. Studies of Korean survival auditions [49–51] have emphasized the importance of competitiveness, fairness, and participation and shown that viewers’ trust in fairness directly affects the success of a program. For example, the *Produce 101* franchise, despite its initial success, was terminated because of fairness issues in viewers’ voting, leading to legal trouble and the disbanding of idol groups [52]. This illustrates that fairness is key to the survival of cultural products.

This study defines the fairness meme more precisely and explores its relationship with related concepts. Fairness encompasses multiple sub-concepts and is linked to an expectation of compliance with societal norms [53,54]. Key fairness criteria include process, outcomes, and expectations [55,56]. We argue that the sub-elements of fairness, namely, audience empowerment, experts’ involvement, fair rewards, and career opportunities, affect viewers’ perceptions of fairness in survival audition programs.

Our research model examines whether these four sub-elements, especially audience involvement, influence the continued success of survival audition programs. Viewers serve as evaluators and overseers to ensure adherence to fairness norms. In the following sections, we introduce our hypotheses based on these fairness norms.

### Audience empowerment and the survival of survival audition programs

First, we posit that survival audition programs that empower the audience as primary participants are more likely to be successful than those that treat the audience as passive observers. The audience played a peripheral role in the early versions of survival audition programs, reacting passively to contestants’ performances. However, as the format of survival auditions evolved, the audience’s role also evolved, and became more central, as an evaluator. Moreover, advancements in telecommunications and the internet empowered home and studio audiences to act as evaluators through telephone, text messaging, and online voting mechanisms. Gunter [57] examined survival audition programs and highlighted the substantial effect of studio and home audience participation as evaluators on the success of these programs.

Empowering the audience considerably contributes to the perceived fairness of a program among the audience. Specifically, when the audience is empowered through direct engagement in the evaluation process, it enhances the perception of procedural justice—an aspect of fairness that deals with the impartiality of the process. Locke and Schweiger [58] posited that stakeholders’ involvement in decision making increases the perception of procedural justice irrespective of the decision outcomes. Additionally, the literature on exit, voice, and loyalty [59–62] has shown that granting the audience a say in decision making may forestall their departure by increasing their perception of fairness. Multiple forms of interaction have been designed to satisfy the fairness perceptions of the public, who act as both viewers and evaluators of audition programs, by actively promoting voting participation [50].

According to Choi [63], *Superstar K* is considered Korea’s most successful survival audition program. It aired for eight seasons over seven years and holds the record for the highest viewership rating in the history of cable channels. The author also highlights that live auditions were broadcast as the show progressed. In the final stage of the program, viewer participation became crucial through text voting (accounting for 60% of the total score) and internet voting (accounting for 10% and was conducted in advance). This active engagement of viewers through voting considerably contributed to *Superstar K*’s success and longevity. In this context, empowering the audience as evaluators may positively influence the longevity of a survival audition program by increasing the audience’s perception of fairness. Consequently, we propose the following hypothesis:

Hypothesis 1: The meme of audience empowerment positively affects the success of survival audition TV programs.

### Expert involvement and the survival of survival audition programs

Some survival auditions actively involve experts, who are called coaches, trainers, mentors, or masters. Experts are different from judges; judges evaluate participants’ performances, whereas experts support, train, and mentor the contestants in their journey in the survival audition programs. Although celebrity judges first appeared in survival audition programs in the mid-1970s, involving experts in audition programs is more recent.

Counter-intuitively, we argue that there is a negative relationship between experts’ involvement and the longevity of survival audition programs from a fairness perspective. This unexpected adverse effect of experts’ involvement demonstrates the underlying logic of our research model that focuses on the meme of fairness. On the surface, it may seem that experts’ involvement positively contributes to the longevity of audition programs because it results in the growth of contestants’ skills, ability, and performance quality over multiple rounds of the competition. However, from a fairness perspective, experts’ involvement may be a double-edged sword because the coaching, mentoring, and assistance provided by experts to contestants may harm the core meme of fair competition in survival audition programs.

Rather than competing purely on the basis of participants’ ability and efforts, winning shows based on expert assistance may be considered unfair. As most contestants in a survival audition are amateurs who are talented but unable to attract the attention they deserve from the public, the intervention of experts may seriously undermine the meme of fair competition among such amateurs.

Park [64] and Kim [65] investigated the fairness of experts in survival auditions and identified flaws in the objectivity of the guidance, screening, and mentoring process, which casts doubt on the validity of fairness claims. Park [66] highlighted issues in *K-Pop Star*, a popular survival audition program, noting that the role of judges was marked by inconsistencies in evaluations, fluctuating evaluation standards, and subjective opinions, negatively affecting viewers’ fairness perceptions. Therefore, we propose that experts’ involvement negatively influences viewers’ perceptions of fairness in the survival audition process. Specifically, when an expert intervenes to give advice or assess contestants, it gives rise to the issue of whether viewers concur or disagree with the expert’s assessment. Put differently, viewers’ agreement with the expert’s actions and evaluations can influence the perceived fairness of a show. This process necessitates building a consensus by connecting individual-level propriety and group-level validity. However, if viewers’ opinions do not match the experts’ judgment, it can undermine the perceptions of fairness and the legitimacy of the show [67]. Therefore, including experts in a survival audition program is likely to negatively affect viewers’ perceptions of the program’s adherence to fairness norms, thereby compromising the program’s success. Therefore, we propose the following hypothesis.

Hypothesis 2: The meme of experts’ involvement negatively affects the success of survival audition TV programs.

### Fair reward and the survival of survival audition programs

Providing equitable rewards to contestants who are rated highly is also likely to affect the success of survival audition programs. Audience empowerment is associated with procedural justice, whereas fair compensation for the winning contestants is associated with distributive justice. Distributive justice is related to the balance between performance and reward; it has been shown that people seek to achieve a perceived equilibrium or fairness between outputs and rewards [68–70]. The payment of rewards in a survival audition program signifies that talented participants receive fair and equitable treatment based on their performance.

In most survival auditions, the audience perceives distributive justice when they witness previously unknown contestants progress through multiple rounds of the competition and emerge as a star entertainer through their ability and skills. Therefore, the perception of distributive justice is validated when the audience is assured that high-performing contestants receive rewards that are commensurate with their performance. In *Star Search*, a popular survival audition program in the 1980s, the $100,000 prize given to the final winner was a key factor that contributed to it becoming the longest-running survival audition program in the history of the broadcasting industry [57].

In this context, we argue that the reward structure of survival auditions considerably influences viewers’ perceptions of fairness in such shows and their longevity. Research on survival audition programs has shown that the prize money awarded by these programs have gone up to $1 million; however, concerns have been raised about excessive competition for the prize money [71] Moreover, some survival audition programs have failed to deliver the promised prize money after the conclusion of the program, which seriously undermines the show’s credibility [72,73]. If fair compensation is not provided to contestants of survival audition programs, it can negatively affect the motivation of potential contestants to participate in such programs in the future and the motivation of viewers to watch these programs. Consequently, from a distributive justice standpoint, we propose the following hypothesis.

Hypothesis 3: The meme of fair rewards positively affects the success of survival audition TV programs.

### Career opportunity and the survival of survival audition programs

Which show is more likely to succeed? A survival audition program that offers an opportunity to enter show business to the winner (the last survivor standing) or a program that does not? A survival audition program is a cultural commodity that is planned and produced by broadcasting companies for economic performance. However, it also has a social commitment to provide contestants a legitimate opportunity to achieve their dreams through verification, such as the talent search process and evaluation. The public and viewers do not merely enjoy and appraise contestants’ performances; they also anticipate that the winners, who have undergone verification, will be granted the chance to make a career in the showbiz industry [15,16].

Some survival audition programs offer contestants who attain high evaluations various opportunities to embark on a professional entertainment career. In contrast, others only offer prizes and provide no guidance for the career development of audition winners. The meme of offering a career opportunity is crucial, as the primary objective of a survival audition program is to unearth fresh talent and help them become a star [57]. In this context, survival auditions that fail to provide a career opportunity to the winners may be perceived as a fictional show lacking realism.

One critical aspect of distributive justice is fairness between performance and compensation [68]. Hence, the ultimate compensation for contestants in a survival audition program is not limited to the prize money but becoming a successful professional entertainer. Winning the audition show is the gateway to real-world success in the entertainment industry. Thus, if the final winner is granted an opportunity to become an entertainer, the perception of distributive justice is validated. Using case studies, researchers have proposed strategies for survival audition programs. According to Park [71], audition programs, such as *Hello Rookie* that aims to discover and support indie bands, underscore the importance of creativity. *Hello Rookie* winners get a chance to perform at major music festivals, such as the Pentaport Rock Festival and the Grand Mint Festival, during the final stage of the competition. However, many survival audition programs are limited to discovering future stars; there is a recognized issue regarding the need to give contestants a career opportunity. The absence of career opportunities to contestants in survival auditions, particularly the winners, has been extensively documented. This phenomenon substantially influences the motivation of prospective contestants to compete in such programs [71] and the motivation of viewers to watch them [74]. In this context, survival audition programs that do not officially provide an opportunity to enter show business are likely to be perceived as unfair by the public and viewers. Therefore, we propose the following hypothesis.

Hypothesis 4: The meme of career opportunity positively affects the success of survival audition TV programs.

## Method

Following Rahi’s [75] research strategy for quantitative methods, we used archival and historical analyses in this study. We used archival data to code dependent variables, such as the scheduling of follow-up survival audition programs, and control variables, such as broadcasting channels, number of episodes, licensing, and political change. Additionally, the success of survival audition programs was examined from a historical perspective, analyzing how this phenomenon emerged, evolved, and expanded, while identifying the key factors influencing its survival and spread.

### The empirical setting: The history of TV survival audition programs in Korea

*Akdong Club* is the archetype of Korean survival audition TV programs; it was broadcast on MBC from 2001 to 2002. The program had two parts: the first half was a talent show audition to recruit singers, whereas the second half was a reality show that followed the growth and debut of the selected singers. Although *Akdong Club* featured many core memes of survival auditions, it was primarily a reality show that focused on developing underperforming students.

**Fig 1:**
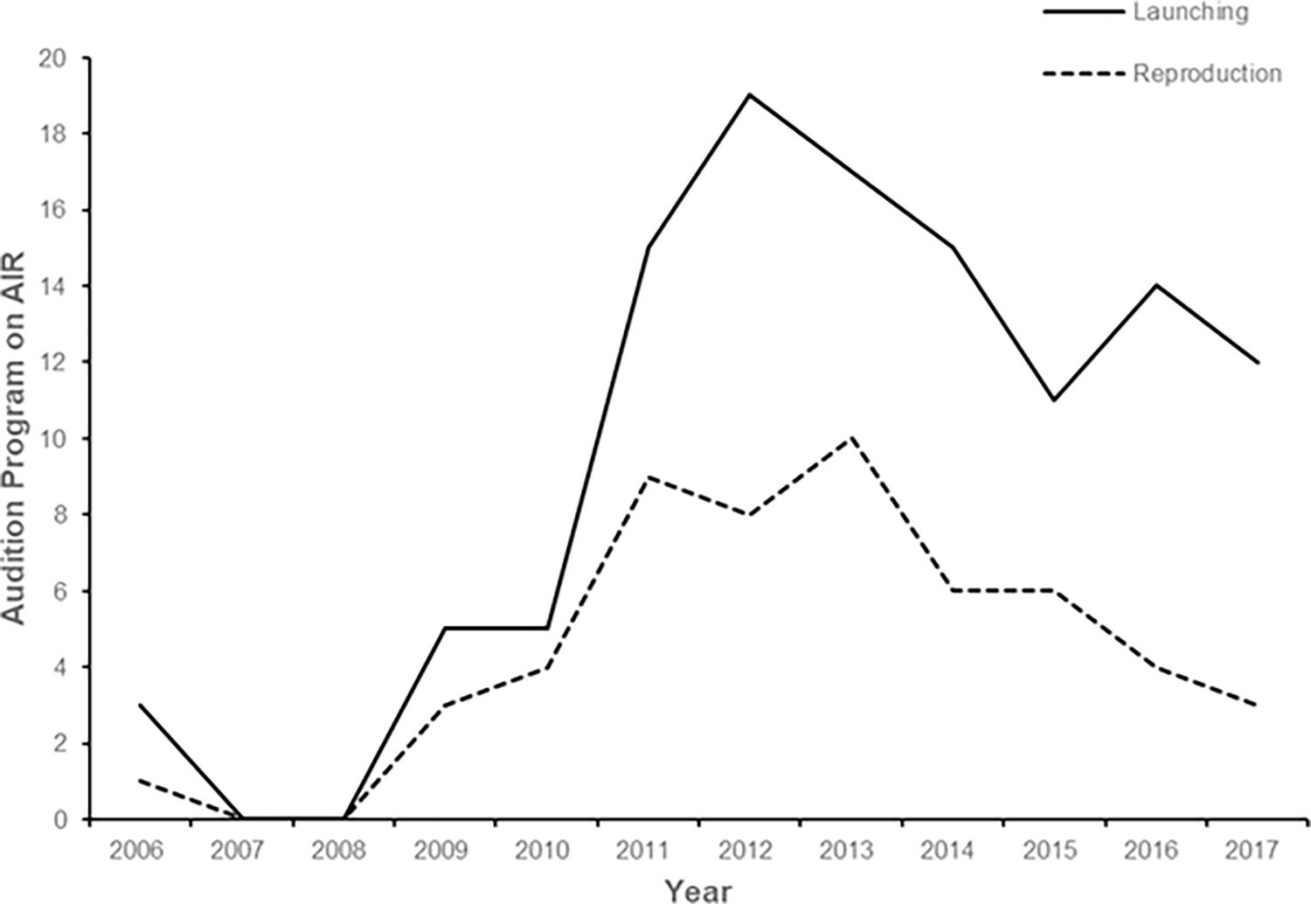
Korean survival audition TV programs on air.

The first full-fledged survival audition program in Korea was *Superstar Survival*, a Korean version of *Star Search*, which aired on SBS in 2006. Contestants competed in multiple rounds of singing, dancing, and acting. However, like the early American survival programs, *Superstar Survival* ended after the first season. The explosion of survival audition programs in Korea coincided with the launch of numerous cable TV channels, particularly the music channel Mnet. In 2009, Mnet introduced the popular *Superstar K* franchise, which achieved a record viewership of 18.1%—the highest in Korean cable broadcasting history; Mnet’s usual viewership rating was 1.6%–1.7%. *Superstar K* ran from 2009 to 2016 and became the standard for subsequent Korean survival audition programs, inspiring other franchises, such as *Show Me the Money* and *K-Pop Star*. Since 2008, many survival audition programs have aired in South Korea, but not all have enjoyed a long run or been successfully replicated (Fig 1). This study aims to investigate the factors contributing to the endurance and replication of these programs by focusing on the role of memes.

### Research design and data collection

Before addressing the main topic, it is important to distinguish between “survival programs” and “audition program” formats. In a survival program, contestants have to perform tasks throughout the duration (one season) of a show and face elimination if they fail to complete a task. Typically, these programs have a reality TV format and show the competition in a documentary style, for example, *Survival* and *Big Brother* by Endemol in the Netherlands. In contrast, an audition program identifies winners through singing, dancing, or other talent-related competitions, with participants competing for monetary rewards or prizes; for example, *American Idol* [76].

Most audition programs follow a survival format, although some one-time audition programs do not, and some survival programs deviate from conventional audition program concepts. In this study, we define a survival audition program as a format in which participants undergo talent-based tasks throughout the duration of a show. This leads to both eliminations and a final winner, who receives prize money or an opportunity to demonstrate their talent professionally.

We used data from 116 survival audition programs broadcast in Korea from 2006 to 2017. An initial keyword search for “survival” and “audition” conducted across five terrestrial broadcasters, 16 cable broadcasters, and four general programming broadcasters in Korea yielded 124 survival programs that were (i) produced by Korean broadcasters, (ii) aired more than twice, and (iii) met the audition program criteria mentioned above. Programs with insufficient information or that aired sporadically were excluded, resulting in a final dataset of 116 programs for analysis.

The analysis focused on survival audition programs, compiling details, such as broadcast channel, genre, duration, program specifics, and cast information (judges, contestants, show host, mentors, and coaches). Additionally, data on support methods, prizes, and benefits were gathered from the official websites and press releases of 25 broadcasters and the annual video industry white paper published by Korea Creative Content Agency. Four coders reviewed the data for accuracy and consistency to ensure intercoder reliability and coded cultural elements following predefined rules.

Table 1 shows that of the 116 programs examined, 63 are music-related (54.31%, such as *Superstar K* and *Show Me the Money)*, 12 are startup/entrepreneurship-related (10.34%, such as *Golden Octagon*), eight are fashion model selection programs (6.90%, such as *Challenge Supermodel*), eight are art/design-related (6.90%), and seven are restaurant-related (6.09%, such as *Master Chef Korea*).

**Table 1:** Data summery and description.

Variables	Classification	N	%
Category	Music (i.e., Vocal, Hip-Hop, Rock Band, and Etc.)	63	54.31
	Startup and Entrepreneurship	12	10.34
	Fashion Model	8	6.90
	Art and Design	8	6.90
	Chef and Cooking	7	6.03
	Content Creation	4	3.45
	Dance	2	1.72
	Others (i.e., Photograph, Programing, Acting, and Etc.)	12	10.34
	Total	116	100.00
Period	1 Days ~ 50 Days	28	24.14
	51 Days ~ 100 Days	71	61.21
	101 Days ~ 150 Days	17	14.66
	Total	116	100.00
Episodes	EP 1	1	0.86
	EP 2 ~ EP 10	32	27.59
	EP 11 ~ EP 20	71	61.21
	EP 21 ~ EP 40	12	10.34
	Total	116	100.00
Viewing Rates	0.00% ~ 1.00%	54	46.55
	1.01% ~ 5.00%	45	38.79
	5.01% ~ 10.00%	7	6.03
	10.01% ~	10	8.62
	Total	116	100.00

Regarding the duration of airing, 71 shows (61.21%) were on air for 51 to 100 days and 17 shows (14.66%) were on air daily, while 28 shows (24.14%) were on air for an unspecified duration. More than half of the programs lasted for 51 to 100 days, approximately 1.5 to 3 months.

Regarding production costs and number of episodes, one show (0.86%) aired only one episode, 10 shows (27.59%) aired 2 to 10 episodes, 71 shows (61.21%) aired 20 episodes, and 12 shows (10.34%) aired between 21 and 40 episodes. The varying number of episodes and airing schedules, spanning 2 to 5 months, reflect broadcasting companies’ preferences and highlight the importance of evaluating the duration and frequency of viewer engagement.

Regarding viewership ratings, which is a key measure of success, 46.55% of the shows had a rating of 0.00%–1.00%, 38.79% had a rating of 1.01%–5.00%, and 8.62% had a rating of above 10.01%. These statistics underscore the challenges faced by survival audition programs in achieving success, especially when compared with the average cable viewership rating of 13.50% during the same period.

### Measurements: Dependent variable

Our dependent variable is the reproduction of a TV survival audition program. Unlike tangible goods, cultural goods are idiosyncratic objects in that they cannot be reproduced in the strict sense, except a rerun. Therefore, in this study, we define the reproduction of survival audition program as the spin-off of the same format in a franchise rather than a rerun of the same content. We consider that a survival audition program can be reproduced in the following two ways. First, we include a sequel of the focal survival audition program with the same format or an extension of the earlier series (i.e., *Superstar K—Superstar K2~ K7*, *K-Pop Star—K-Pop Star K2~K6*). For example, *Star Wars Episode V*: *The Empire Strikes Back* is an example of a sequel in the *Star Wars* movie franchise, while *Rogue One*: *A Star Wars Story* is a spin-off of the original film. Second, we include a spin-off of the focal survival audition program that retains one or more aspects (i.e., theme, topics, characters, events) of the original format (e.g., *Voice of Korea—Voice of Korea Kids*). We assigned this variable a value of 1 if a survival audition TV program satisfies either of these conditions, and 0 otherwise.


$$Reproduction=\left\{ \begin{array}{c} 1\;if\;a\;sequel\;or\;spin-off\;is\;made\\ 0\;otherwise \end{array}\right.$$

### Measurements: Independent variables

It is important to accurately measure the influence of cultural memes on the reproduction of cultural goods. Few studies have endeavored to conceptually delineate and quantify the cultural and normative codes embedded in cultural goods; hence, it is challenging to measure memes. Sperber [77, pp.103] posited, “The cultural significance of a public production should not be measured solely by the quantity of copies present in the environment but by their impact on individuals’ perceptions.” McNamara [78] too highlighted the importance of a meme’s influence over its quantity, arguing that the apparent difficulty in the measurement of memes stems from the lack of an authoritative definition of a meme. Acknowledging the limitations in the literature regarding meme measurement, Kim and Shin [79] devised a qualitative approach to appraise cultural phenomena as an alternative method. Cultural phenomena, especially factors, such as cultural and normative codes, are qualitative variables that cannot be represented numerically [80,81]. Nonetheless, the quantitative aspect could be used to generalize the qualitative findings [82]. Following Kim and Shin [79], who measured cultural and normative codes in survival audition programs using dummy variables [83], we measure memes in this study in the following way.

The first independent variable of this study is *Audience Empowerment*. Studies on survival audition programs have emphasized the effects of evaluating contestants’ performance by studio and home audiences [57]. We coded this variable as 1 if the focal program allowed the audience to vote on contestants’ performance (e.g., viewers can vote through the website, SMS, etc.), and 0 otherwise.


$$Audience\;Empowerment=\left\{ \begin{array}{c} 1\;if\;the\;audience\;votes\;on\;performance\\ 0\;otherwise \end{array}\right.$$

We measured our second independent variable, *Expert Involvement*, by the involvement of one or more experts in the survival audition program. We analyzed all of the survival audition programs and found that experts can participate in the following two ways. First, they participate by engaging in the survival audition process as coaches, trainers, mentors, or masters providing support, training, and advice, which affects contestants’ performance and skill level and influences the competition. Second, they directly or indirectly influence contestants’ survival decisions, which affects viewers’ perception of fairness. Hence, we assigned this variable a value of 1 if experts participate in a survival audition program, and 0 otherwise.


$$Expert\;Involvement=\left\{ \begin{array}{c} 1\;if\;experts\;guide\;contestants\;during\;the\;program\\ 0\;otherwise \end{array}\right.$$

The third independent variable, *Fair Reward*, is also a binary variable. We verified whether there was a provision of a cash prize for the winners of survival audition programs conducted in Korea. However, variations in the genre and nature of the audition program resulted in differences in the cash amount awarded. Directly measuring the amount was challenging as it represented the size of compensation. Consequently, we assigned this variable a value of 1 if the focal program offered a cash prize for the final winner, and 0 otherwise.


$$Fair\;Reward=\left\{ \begin{array}{c} 1\;if\;there\;is\;a\;cash\;prize\;for\;the\;final\;winner\\ 0\;otherwise \end{array}\right.$$

The last independent variable is *Career Opportunity*. This is crucial because the main purpose of a survival audition program is to offer a career opportunity to talented contestants. Some survival audition programs in which contestants are aspiring entrepreneurs may give a cash prize or investment in the form of seed money, enabling contestants to launch a business based on their ideas and talents. However, direct career opportunities, such as giving a record deal to a singer, are not offered. We posit that the variations in qualitative attributes, specifically the assurance of an opportunity to sing or act, affect a program’s reproducibility. Therefore, we coded this variable as 1 if the focal survival audition program offers a contract (i.e., an official recording contract or debut concert) to the final winner; and 0 otherwise.


$$Career\;Opportunity=\left\{ \begin{array}{c} 1\;if\;a\;performance\;contract\;is\;obtained\;after\;the\;audition\;program\\ 0\;otherwise \end{array}\right.$$

### Measurements: Control variables

#### Broadcasting channel

We controlled for the potential effect of broadcasting channels on the reproduction of survival audition programs. In the Korean TV broadcasting industry, channels are classified into three categories: terrestrial, cable, and general programming broadcasting. We created dummy variables for all three categories (*Terrestrial Broadcasting*, *Cable Broadcasting*, *and General Programming Broadcasting*) and included them in our research model.

#### License acquisition

Studies have shown that the acquisition of a license may influence the success and recognition of cultural content. We controlled for the potential effects of the acquisition of a license by creating a dummy variable (*License*). We coded it as 1 if the focal program acquired a legal license from its original production company, and 0 otherwise.

#### Application openness

The most important component of a survival audition program is the contestants because the underlying logic of this cultural good is the search for unknown talents who could become a star. If the contestants are not talented or skilled enough to draw the attention of the audience, the survival audition program is likely to have low viewership. Therefore, the process of recruiting contestants is important. We divided the process of recruitment/application into open and closed. Next, we controlled for the potential effects of these two types of recruitment/application by using a dummy variable, which was coded as 1 if a program had an open application for participation, and 0 otherwise.

#### Ratio of duration to episodes

The literature has shown that the duration and number of episodes can interact with each other and influence the success of cultural goods. Thus, we controlled for their potential effects using a variable (*Duration*/*Episodes*), which was measured by counting the total period that a survival audition program was broadcast in days and dividing it by the number of episodes aired.

#### Political change

The literature on the cultural industry has shown that the political environment can affect the production and consumption of cultural goods. In the history of the Korean cultural industry, the period of military rule in the 1980s was marked by severe abuse of culture to distract the public’s attention from political and social problems. The military government vigorously implemented the so-called “3S policy” to “dumb down” Korean people by diverting their attention to sports, sex, and screens [84]. 2017 was also a politically turbulent year, when the 11th president of Korea was impeached, the first time since the founding of the republic. Consequently, Korea’s political, cultural, social, and economic environments were highly volatile in 2017. Hence, we controlled for the potential effect of political change by including a period dummy for 2017.

#### Population size

To control for the potential influence of demographic factors that may affect the production, consumption, success, and reproduction of cultural goods, population was included in the model as a control variable. The population of South Korea from 2006 to 2017 was divided by 10,000 and then transformed into a natural logarithm.

#### Competition

The term “cultural industry” presumes the presence of a market mechanism, such as competition in the production, distribution, and consumption of cultural goods [85]. Therefore, cultural goods are often believed to compete with other cultural goods in the market. Multiple survival audition programs that air during the same period will fiercely compete with each other for media exposure, viewers’ attention, and potential contestants. We controlled for the potential effects of competition, which was measured by counting the number of survival audition programs that aired each year.

To address concerns about data collection and variable measurement, we took the following steps to address coder subjectivity and intercoder reliability (ICR). Following the guidelines of Lombard et al. [86] and MacPhail et al. [87], we reviewed studies on qualitative data collection and measurement. Additionally, we applied the recommendations of O’Connor and Joffe [88] to mitigate ICR issues. Four master’s-level coders were recruited, and their results were compared and verified to reduce subjectivity.

### Statistical estimation

We used probit regression for the statistical estimation. Given the binary nature of our dependent variable, we estimated the likelihood of reproduction or discontinuation of survival audition programs during the study period. We did not use survival analysis in this study, which examines the influence of cultural memes on the replication of cultural goods, for two reasons. First, survival analysis predicts the probability of occurrence of an event over time by observing a single entity. After one program comes to an end, subsequent series/shows, though similar, are not the same entity. Second, survival analysis typically deals with one-time events, such as death or accidents, whereas survival audition programs can run for multiple seasons, making traditional survival analysis unsuitable. Thus, after careful consideration, we used probit regression with a binary dependent variable to estimate the causal effects of the four independent variables on the reproduction of survival audition programs.

## Results

Table 2 presents the descriptive statistics of all the variables in our model. The average of *Reproduction* is 0.4655, which indicates that half of the survival auditions ran for multiple seasons while half were discontinued after just one season.

**Table 2:** Descriptive statistics.

#	Variables	Mean	S.D.	1	2	3	4	5	6
1	Reproduction	.47	.50	1.00					
2	Audience Empowerment	.22	.41	-0.03	1.00				
3	Expert Involvement	.70	.46	0.16	-0.80	1.00			
4	Fair Reward	.16	.36	0.22	-0.22	0.28	1.00		
5	Career Opportunity	.86	.35	0.17	0.21	-0.21	0.10	1.00	
6	Terrestrial Broadcasting	6.38	.93	0.19	0.09	-0.11	-0.06	-0.01	1.00
7	Cable Broadcasting	5.24	2.38	-0.19	-0.07	-0.09	-0.18	-0.33	-0.04
8	License	.10	.31	-0.15	-0.04	-0.21	-0.15	-0.11	-0.04
9	Application Openness	13.97	4.15	-0.05	0.01	-0.03	0.14	0.02	-0.05
10	Duration/Episodes	.65	.48	0.11	0.17	-0.13	-0.28	-0.14	0.08
11	Politics Change	.28	.49	-0.12	0.17	-0.08	-0.10	0.13	0.08
12	Population Size	.77	.42	0.27	0.09	-0.14	0.18	0.73	0.04
13	Competition	.74	.44	0.12	-0.07	0.13	-0.02	-0.24	0.08
#	Variables		7	8	9	10	11	12	13
1	Reproduction								
2	Audience Empowerment								
3	Expert Involvement								
4	Fair Reward								
5	Career Opportunity								
6	Terrestrial Broadcasting								
7	Cable Broadcasting		1.00						
8	License		0.28	1.00					
9	Application Openness		-0.21	0.10	1.00				
10	Duration/Episodes		-0.11	-0.06	-0.01	1.00			
11	Politics Change		-0.21	-0.15	-0.11	-0.04	1.00		
12	Population Size		-0.06	-0.09	-0.28	-0.02	0.31	1.00	
13	Competition		-0.03	0.14	0.02	-0.05	-0.16	0.46	1.00

Table 2 also shows the correlations between the variables. None of the correlation values are statistically significant. We also check the variance inflation factor (VIF) to confirm that multicollinearity is not an issue in our statistical models. The average VIF is 1.92, with the highest value of 3.75 for *Cable Broadcasting*. Generally, VIF values below 10 are considered statistically acceptable; all of the variables used in this study are free of multicollinearity [89,90].

Table 3 presents the probit regression results. Model 1 is the baseline model and includes only the control variables. Some of the control variables were found to have a statistically significant effect on the reproduction of survival audition programs. Both *Cable Broadcasting* and *Duration/Episodes* were positively correlated with the reproduction of survival auditions. Because cable broadcasting channels specialize in specific TV program genres, such as music survival auditions, it may have positively affected the likelihood of reproduction. Also, we can infer that the ratio of broadcast time to the number of episodes had a significant effect on the reproduction of survival audition programs because it is positively related to media exposure and viewership.

**Table 3:** Probit regression: Predicting survival audition reproduction.

Variable	Model 1		Model 2		Model 3		Model 4		Model 5		Model 6	
Audience Empowerment			0.72	*							1.18	***
			(0.30)								(0.35)	
Expert Involvement					-0.48	^†^					-0.68	*
					(0.28)						(0.32)	
Fair Reward							1.05	*			1.57	**
							(0.50)				(0.57)	
Career Opportunity									0.45	^†^	0.76	*
									(0.30)		(0.34)	
Terrestrial Broadcasting	1.04	^†^	0.97	*	1.30	*	1.20	^†^	1.01		1.61	*
	(0.64)		(0.66)		(0.67)		(0.65)		(0.65)		(0.76)	
Cable Broadcasting	1.32	*	1.35	*	1.55	*	1.48	*	1.28	*	1.92	**
	(0.61)		(0.64)		(0.63)		(0.62)		(0.62)		(0.73)	
License	0.71	^†^	0.96	*	0.65	^†^	0.60		0.73	^†^	0.91	*
	(0.38)		(0.41)		(0.39)		(0.39)		(0.39)		(0.43)	
Application Openness	0.75	^†^	0.84	*	0.86	*	-0.15		0.84	*	-0.13	
	(0.41)		(0.42)		(0.41)		(0.60)		(0.41)		(0.64)	
Duration/Episodes	0.38	*	0.41		0.40	*	0.36	*	0.39	*	0.47	*
	(0.16)		(0.17)		(0.16)		(0.16)		(0.17)		(0.19)	
Politics Change	-0.15		-0.20		0.03		-0.08		-0.17		0.18	
	(0.51)		(0.53)		(0.52)		(0.53)		(0.52)		(0.60)	
Population Size	1.96		-6.89		6.76		6.63		-1.24		-6.24	
	(21.70)		(22.57)		(22.11)		(22.40)		(21.79)		(24.12)	
Competition	-0.02		-0.02		-0.01		-0.03		-0.01		-0.01	
	(0.04)		(0.04)		(0.04)		(0.04)		(0.04)		(0.04)	
Constant	-20.85		53.87		-62.17		-60.58		5.82		46.10	
	(184.56)		(191.98)		(188.16)		(190.55)		(185.28)		(205.04)	
Observation	116		116		116		116		116		116	
LR Chi-Square	22.19		28.11		25.21		27.10		24.42		45.38	

Values in parentheses are standard errors.

^†^ p < .10

* p < .05

** p < .01^,^

*** p < .001.

In Models 2–6, all four independent variables consistently show a statistically significant effect on the reproduction of survival audition programs in a highly robust manner. In Model 2, the main effect of *Audience Empowerment* is positive and significant, supporting Hypothesis 1. In Model 3, *Expert Involvement* during survival auditions is negatively correlated with the likelihood of reproduction, suggesting that the audience considers competition based on experts’ advice and assistance to be unfair. Model 4 supports Hypothesis 3, which predicts a positive relationship between *Fair Reward* for the winner and the reproduction of survival audition programs. This result indicates that the audience considers it fair that the winner is rewarded for their outstanding performance. In Model 5, the effect of *Career Opportunity*, a core characteristic of survival auditions, is positive and weakly significant. Finally, in Model 6, all of the independent variables remain strongly significant in the entire model. The results support all four hypotheses.

We used various statistical methodologies and measures for the dependent variable to enhance the statistical robustness of our model. Logistic regression is commonly used for binary dependent variables, in addition to probit analysis, which is based on normal distribution [81,89]. Table 4 presents the logistic regression results of Models 7–11 on the same research model. Models 12–16 demonstrate the results derived from measuring the number of reproductions of each survival audition program as the dependent variable. Consistent with the previous results, the results show that all of the variables, except *Career Opportunity*, support the hypothesized relationships. Nevertheless, there are disparities in the confidence levels.

**Table 4:** Additional analysis: Predicting survival audition reproduction.

Variable	Logistic Regression											Y = # of Reproduction									
	Model 7		Model 8		Model 9		Model 10		Model 11			Model 12		Model 13		Model 14		Model 15		Model 16	
Audience Empowerment	1.19	*							2.03	***		0.62	*							0.95	**
	(0.52)								(0.62)			(0.30)								(0.29)	
Expert Involvement			-0.82	^†^					-1.21	*				-0.68	*					-0.80	**
			(0.46)						(0.57)					(0.29)						(0.28)	
Fair Reward					1.71	*			2.65	**						1.18	*			1.35	**
					(0.86)				(0.99)							(0.47)				(0.46)	
Career Opportunity							0.71		1.25	*								0.24		0.40	**
							(0.50)		(0.59)									(0.33)		(0.30)	
Terrestrial Broadcasting	1.66		2.31	^†^	2.10	^†^	1.74		2.83	*		0.52		1.03	^†^	0.94	^†^	0.66		1.03	^†^
	(1.22)		(1.26)		(1.22)		(1.19)		(1.43)			(0.58)		(0.59)		(0.58)		(0.59)		(0.56)	
Cable Broadcasting	2.33	*	2.71	*	2.57	*	2.16	^†^	3.34	*		0.96	^†^	1.32	*	1.26	*	1.00	^†^	1.40	**
	(1.19)		(1.22)		(1.18)		(1.16)		(1.38)			(0.54)		(0.55)		(0.54)		(0.55)		(0.52)	
License	1.53	*	1.05		0.96		1.16	^†^	1.47	*		0.04		-0.26		-0.29		-0.16		-0.15	
	(0.68)		(0.66)		(0.66)		(0.65)		(0.72)			(0.41)		(0.40)		(0.40)		(0.40)		(0.38)	
Application Openness	1.36	*	1.48	*	-0.25		1.38	*	-0.14			0.63		0.74	^†^	-0.45		0.62		-0.16	
	(0.69)		(0.70)		(1.02)		(0.69)		(1.11)			(0.43)		(0.43)		(0.58)		(0.44)		(0.56)	
Duration/Episodes	0.67	*	0.67	*	0.61	*	0.64		0.82	*		0.25	^†^	0.30	*	0.24	*	0.26	^†^	0.23	^†^
	(0.30)		(0.29)		(0.30)		(0.29)		(0.33)			(0.15)		(0.15)		(0.14)		(0.15)		(0.14)	
Politics Change	-0.30		0.09		-0.07		-0.27		0.27			0.06		0.28		0.22		0.06		0.40	
	(0.91)		(0.91)		(0.89)		(0.89)		(1.08)			(0.53)		(0.54)		(0.53)		(0.54)		(0.51)	
Population Size	-11.16		12.69		10.56		-0.91		-5.74			-32.58		-19.79		-22.27		-26.95		-27.56	
	(37.45)		(36.57)		(37.52)		(36.11)		(40.48)			(24.41)		(24.18)		(23.98)		(24.64)		(22.89)	
Competition	-0.04		-0.02		-0.04		-0.02		-0.02			-0.01		0.01		-0.01		0.00		0.01	
	(0.06)		(0.06)		(0.06)		(0.06)		(0.07)			(0.04)		(0.04)		(0.04)		(0.04)		(0.04)	
Constant	86.94		-115.97		-97.01		-0.01		36.64			275.40		166.01		187.76		227.42		231.47	
	(318.47)		(311.20)		(319.25)		(307.12)		(344.24)			(207.73)		(205.80)		(204.09)		(209.64)		(194.81)	
Observation	116		116		116		116		116			116		116		116		116		116	
LR Chi-Square	27.85		25.33		26.92		24.07		45.46												
R-squared												0.1376		0.1468		0.1539		0.1086		0.2710	
Adj R-squared												0.0644		0.0743		0.0821		0.0329		0.1861	

Values in parentheses are standard errors.

^†^ p < .10

* p < .05

** p < .01^,^

*** p < .001.

## Discussion and conclusion

This study investigates the evolutionary dynamics of cultural goods by empirically examining the influence of cultural memes on the renewal of survival audition programs in the Korean TV broadcasting industry, with a focus on fairness. The findings indicate that cultural memes embodying fairness norms, namely, audience empowerment, experts’ involvement, fair rewards, and career opportunities, significantly affect the continuation of survival audition programs. These findings offer valuable insights for both industry practitioners and academic scholars in cultural industries.

Practically, the results underscore the need to implement tailored strategies depending on the type of cultural goods. Unlike the manufacturing sector, where efficiency is a competitive advantage, creativity is a competitive advantage in the cultural industries [25]. Differentiation through creativity is critical in the early stages of development of cultural goods. However, industry practitioners should focus on broad audience acceptance for successful goods, such as survival audition programs. The findings of this study underscore the importance of fairness memes as a fundamental design element for such goods. Cultural industry professionals must distinguish between goods that aim for immediate popularity and those that aim for sustained success over time.

Theoretically, this study contributes to literature by proposing a meme-based approach to analyze cultural goods. Conventional approaches focusing on the unique characteristics of cultural goods fail to explain macro-level evolutionary dynamics because of the idiosyncratic nature of individual goods. In contrast, a meme-based approach reveals industry-wide evolutionary patterns, as cultural memes such as fairness can transcend the lifespan of individual goods and be transmitted to others [36–38]. The findings of this study show that memes, such as audience empowerment and experts’ involvement, are reproduced by both spin-off programs and other cultural products, suggesting a meme-based framework for understanding cultural evolution.

Finally, this study explores the relationship between abstract norms and the tangible expressions used to evaluate cultural products. Social exchange and equity theories emphasize the importance of fairness in the success and spread of cultural products [58,61,69,70,79]. However, how fairness is perceived and judged in different cultural contexts has been underexplored. Survival audition programs require various elements to ensure fairness, and the findings of this study reveal how these elements affect the audience’s perception of fairness. This study contributes to theory and practice by showing the causal relationship between the perception of fairness and the popularity and spread of cultural products.

This study has the following limitations. First, there are methodological challenges in measuring the cultural and normative codes embedded in survival audition programs, particularly the qualitative information. This study examined how cultural factors and internal program characteristics affect program reproduction. Cultural and normative factors with non-ordinal qualitative attributes were included as independent variables, while program characteristics were treated as nominal variables. Four coders were involved to minimize subjectivity, and intercoder reliability was ensured by reaching a consensus. Although qualitative methods could have been used to analyze individual programs, we focused on identifying shared cultural codes across different survival audition programs using binary measurement for empirical analysis. Future studies should incorporate qualitative methods to explore cultural codes in depth.

Second, the exclusion of potentially relevant market-specific factors. As mentioned earlier, market-related data, such as merchandising sales, advertising revenues, license fees, and production costs, which could affect the reproduction of cultural goods are often undisclosed by broadcasters and production companies. Future studies should explore these market-specific factors, including advertisement revenue and the internal characteristics of cultural goods, to better understand the role of cultural and normative memes in the success, diffusion, and reproduction of various cultural goods, such as movies, music, design, and advertising.

Last, the limitations related to region- and time-specific factors, a common problem in research on the cultural industry. Expanding this research to examine the cultural codes influencing the survival of audition program formats across different regions, such as the United States, the Netherlands, China, Japan, and India, is necessary. However, the cultural context of production and consumption must be considered. Although, some socioeconomic factors, such as economic downturns and political instability, were controlled for in this study, it cannot fully account for all regional and temporal variations. Future studies should provide richer and more comprehensive discussions on the influence of cultural codes in cultural goods across diverse regions and industries.

## Supporting information

S1 File (XLSX)
